# Comparison of Sleep and Attention Metrics Among Nurses Working Shifts on a Forward- vs Backward-Rotating Schedule

**DOI:** 10.1001/jamanetworkopen.2021.29906

**Published:** 2021-10-18

**Authors:** Marco Di Muzio, Giulia Diella, Emanuele Di Simone, Mariella Pazzaglia, Valentina Alfonsi, Luana Novelli, Angelo Cianciulli, Serena Scarpelli, Maurizio Gorgoni, Annamaria Giannini, Michele Ferrara, Fabio Lucidi, Luigi De Gennaro

**Affiliations:** 1 Department of Clinical and Molecular Medicine, University of Rome Sapienza, Rome, Italy; 2 Department of Biomedicine and Prevention, University of Rome Tor Vergata, Rome, Italy; 3 Department of Psychology, University of Rome Sapienza, Rome, Italy; 4 Body and Action Lab, IRCCS Fondazione Santa Lucia, Rome, Italy; 5 Department of Biotechnological and Applied Clinical Sciences, University of L’Aquila, L’Aquila, Italy; 6 Department of Developmental and Social Psychology, University of Rome Sapienza, Rome, Italy

## Abstract

**Importance:**

The association of fast backward-rotating shift work (ie, anticlockwise sequence of afternoon, morning, and night shifts) with subjective and objective measures of sleep-wake quality, daytime attention, and tiredness of health care workers has not yet been established.

**Objective:**

To investigate the association of shift rotation direction with tiredness, sleepiness, and sustained attention among nurses working forward- and backward-rotating shifts.

**Design, Setting, and Participants:**

Data of this cohort study were collected from nurses working at 5 midsized Italian hospitals. The nurses had either a forward-rotating schedule (ie, morning to afternoon to night) and or a backward-rotating schedule (ie, afternoon to morning to night). The data were collected from July 2017 to February 2020. Data analysis was performed from May to October 2020.

**Exposures:**

Participants were working either forward- or backward-rotating schedules, in which the sequence of 3 shifts (morning, afternoon, and night) changed in a clockwise or anticlockwise direction.

**Main Outcomes and Measures:**

Sleep data were collected using the Karolinska Sleepiness Scale and Pittsburgh Sleep Quality Index. Sustained attention was measured using the Psychomotor Vigilance Task. Tiredness was evaluated using the Tiredness Symptom Scale.

**Results:**

A total of 144 nurses (mean [SE] age, 41.3 [0.8] years; 92 women [63.9%]) participated in the study; 80 nurses had forward-rotating schedules, and 64 had backward-rotating schedules. Nurses with irregular sleep-wake patterns due to night shift work had poor sleep quality (46 [57.5%] in forward-rotating schedule group; 37 [57.8%] in backward-rotating schedule group). Nurses working backward-rotating shifts exhibited significantly greater sleepiness (*F*_1,139_ = 41.23; *P* < .001) and cognitive slowing (ie, longer median reaction times; *F*_1,139_ = 42.12; *P* < .001) than those working forward rotations. Importantly, these differences were not affected by age, years of employment, and quality of sleep. Of nurses working on a backward-rotating schedule, 60 (93.8%) reported elevated sleepiness (Karolinska Sleepiness Scale score ≥7) after the night shift. The median reaction time (*F*_1,139_ = 42.12; *P* < .001), 10% fastest reaction time (*F*_1,139_ = 97.07; *P* < .001), minor lapses (*F*_1,139_ = 46.29; *P* < .001), and reaction time distribution (*F*_1,139_ = 60.13; *P* < .001) of nurses on backward-rotating schedules indicated a lower level of vigilance, which is negatively associated with neurobehavioral performance.

**Conclusions and Relevance:**

In this study, both shift rotation models were negatively associated with health and cognitive performance. These findings suggest that forward shift rotation may be more beneficial than backward rotation for several measured performance attentional outcomes and sleepiness. Optimization of shift rotations should be implemented to decrease the combination of the negative outcomes associated with shift work and reduce the potential risk of medical errors in health care systems.

## Key Points

**Question:**

Among hospital staff who work day and night shifts, do sleep and attention metrics vary between those who work forward- vs backward-rotating shifts?

**Findings:**

This cohort study of 144 nurses found that fast forward-rotating shifts for nurses were associated with lower levels of tiredness and sleepiness and higher levels of sustained attention compared with backward-rotating shifts.

**Meaning:**

These findings suggest that optimization of shift rotations for nurses should be implemented to decrease the negative outcomes associated with shift work.

## Teaser

This cohort study examines the association of shift rotation direction with tiredness, sleepiness, and sustained attention among nurses working forward- and backward-rotating shifts.

## Introduction

Within the health care industry, 24-hour rotating shifts for hospital nursing staff are necessary to ensure continuity and high-quality care. Shifts vary owing to numerous factors, including the timing and length of work hours (ie, 8- or 12-hour shifts), fixed or rotating schedules, duration of rotations, and number of consecutive days of night work. A nurse’s adaptability to night shifts is often affected by the speed and direction of the shift rotation.

In the US, slow rotations are becoming more common, allowing workers to adjust their circadian rhythm gradually over a period of 2 to 4 weeks,^1^ whereas in Europe and Japan fast rotations (ie, changing every 3-5 days) are more common, which permits workers to maintain constant circadian rhythms.^2^ The direction of shift rotation (ie, clockwise [forward] or counterclockwise [backward]) also affects workers’ adaptability. In forward-rotating shift (FRS) work, morning shifts are followed by afternoon and then night shifts. In contrast, backward-rotating shifts (BRSs) consist of night shifts followed by afternoon and then morning shifts. It is commonly assumed that forward rotation is easier to adapt to physiologically because the human circadian rhythm tends to move forward, and it is more difficult to fall asleep earlier than the normal bedtime.^3^

Prospective studies^4,5^ suggest that the change from a slowly rotating backward system to a slowly rotating forward system has the greatest effect on night shift workers, in terms of improved sleep quality, decreased fatigue, and fewer attention lapses. Over the long term, these improvements are paralleled by some sleep difficulties during the morning and afternoon shifts.^4,5^ Moreover, studies of shift schedules comparing changes from a slow BRS to a fast FRS revealed improvements in sleep quality and sleep duration after a night shift.^6,7,8^ However, no evidence of change or increased sleep quality and length during the day as a result of the change to forward rotation has been provided thus far.^9,10,11,12,13^ Similarly, no difference in self-rated sleep,^14,15,16^ alertness during the shift,^16^ or driving performance^15,17^ were observed when comparing workers following forward and backward rotation schedules.

For many professions with rotating shifts, large-scale data from shift work have suggested that poor sleep quality is associated with backward rotations.^18^ Less is known about the possible effects of the direction of shift-work rotation among specific hospital personnel. A study^19^ of the effects of clockwise and counterclockwise shift-work rotation in 50 Italian nurses indicated that greater self-reported sleep disturbance and poorer work-life balance were observed among nurses working BRSs than among nurses working FRSs.

Confounding these rotating shift results^19,20,21^ is the structure of the data used, including variations in shift length, shift starting time and/or shift number, and shift direction. Furthermore, some studies used the same participants for both shift rotations, whereas others compared different groups or groups with small sample sizes.^22^ Notably, few studies used an instrument with known validity and/or reliability, and more studies included only qualitative outcome measures. Although each shift schedule has implications for health care and productivity, the preferable shift rotation direction remains unclear.^23^

The current study assessed whether shift rotation direction is associated with changes in both subjective and objective measures by using 2 large samples of nurses working 8-hour FRSs or BRSs. We explored the associations of tiredness and sleepiness and metric changes in vigilance performance with several participant-level factors (ie, age, sex, and years of work experience). We hypothesized that a BRS is associated with increased tiredness, sleepiness, and especially with decreased behavioral measures of sustained attention.

## Methods

This study was performed in accordance with the ethical standards of the Declaration of Helsinki^24^ and was approved by the ethical committee of Sapienza University of Rome. Participation was anonymous and voluntary, and each participant provided written informed consent. This cohort study followed the Strengthening the Reporting of Observational Studies in Epidemiology (STROBE) reporting guideline.

### Participants

The data were obtained from a sample of nurses who were recruited from 5 midsized hospitals in Italy: Policlinico Umberto I (48 nurses), San Giovanni Addolorata (15 nurses), the Sant’Andrea of Rome (17 nurses), San Carlo of Potenza (50 nurses), and San Jacopo of Pistoia (14 nurses). The first 3 hospitals (Policlinico Umberto I, San Giovanni Addolorata, and Sant’Andrea) adopted FRSs, whereas the remaining 2 (San Carlo of Potenza and San Jacopo of Pistoia) adopted BRSs. The data collection started on July 2017 and ended on February 2020. The participants were categorized into 1 of 2 groups according to the direction of shift rotation. The inclusion criteria were the absence of medical and chronic disorders as assessed by a clinical interview, a roster based on the sequence of shifts described in the procedure with no change in shifts for the 3 days under investigation, and no naps during the night shift. Trainees were excluded from this study.

### Measures

#### Pittsburgh Sleep Quality Index

An Italian version of the Pittsburgh Sleep Quality Index (PSQI) was used to evaluate the participants’ sleep quality.^25^ This instrument is a reliable, valid, and standardized self-rating scale designed to assess sleep quality during the previous month.^25^ The questionnaire shows high internal consistency (Cronbach α = 0.835) and good reliability (split-half reliability of 0.85).^26^ The PSQI comprises 19 items divided into 7 subscales that are rated on a 0- to 3-point Likert scale to evaluate the subjective quality of sleep, sleep latency, sleep duration, habitual sleep efficiency, sleep disorders, use of hypnotic drugs, and disorders during the day. The sum of the scores of the 7 components gives the overall score, ranging from 0 to 21, with 0 indicating the absence of difficulty and 21 indicating serious difficulties in all areas. Scores greater than 5 indicate poor sleep quality.^26^

#### Karolinska Sleepiness Scale

An Italian version of the Karolinska Sleepiness Scale (KSS)^27^ was used to evaluate the subjective level of sleepiness in the 5 minutes before administering the questionnaire to participants.^28^ The KSS is a 9-point rating scale ranging from “very alert” to “very sleepy, fighting sleep.” The KSS is a reliable and valid drowsiness indicator that correlates with objective electroencephalogram measures of sleepiness and neurobehavioral performance.^27^

#### Tiredness Symptoms Scale

An Italian version of the Tiredness Symptoms Scale (TSS)^28^ was used to evaluate symptoms of chronic fatigue. The TSS is a dichotomous checklist of 14 physical and emotional symptoms that the participant may experience at the time of evaluation.

#### Psychomotor Vigilance Task

The psychomotor vigilance task (PVT) is a well-established, computerized, simple, cued reaction time (RT) task^29^ that provides the most widely used metrics of sustained attention and sleep loss.^30^ During the PVT, participants are placed in front of a computer screen for 5 minutes and asked to click the left mouse button every time a scrolling timer appears, at irregular intervals, with a random interstimulus interval (from 2 to 100 seconds). In this manner, vigilance must be maintained throughout the 5-minute task. The dependent variables used to quantify PVT include median RT, speed (reciprocal of RT), 10% slowest RT, and 10% fastest RT. Validity and sensitivity of this 5-minute version of the PVT has been shown by independent studies.^31,32,33^

### Procedure

Each participant was studied 3 times: after the morning, afternoon, and night shifts. The rosters were as follows: for FRS, day A was morning (7:00 am to 1:30 pm), day B was afternoon (1:30 pm to 8:00 pm), day C was night (8:00 pm to 7:00 am), day D was dismount (daytime off duty), and day E was rest. For BRS, day A was afternoon (2:00 pm to 8:00 pm), day B was morning (7:00 am to 2:00 pm), day C was night (8:00 pm to 7:00 am), day D was dismount (daytime off duty), and day E was rest.

Hence, all nurses were evaluated after their shift on 3 consecutive days (days A through C). Each testing session (maximum duration, 15 minutes) was conducted in a room without noise or environmental distractions. The nurses were asked to switch off their mobile phones during the session, and no one was allowed to enter, except for the experimenter. The task and questionnaires were administered in a fixed order as follows: KSS, TSS, and PVT. The PSQI was administered only during the first testing session.

### Statistical Analysis

The following dependent variables were considered: TSS and KSS scores and measures of the PVT (median RT, speed, minor lapses [>500 milliseconds RT], major lapses [>1000 milliseconds RT], 10% slowest RT, 10% fastest RT, false starts [premature responses or response times <100 milliseconds], and response time divergence as a measure of variability [ie, dissimilarity of RT probability density functions]). Each dependent variable was analyzed using a repeated measures analysis of covariance, with independent variables of direction of shift rotation (forward or backward) and shift (morning, afternoon, or night), and covariates of PSQI scores, age, and years of work experience. Paired *t* tests were used for post hoc comparisons. The significance level was set at *P* < .05. Data were tested for normality (Kolmogorov-Smirnov test) and homoscedasticity of variance (Bartlett test). We found that PVT did not fit normal distributions (minor and major lapses, false starts, and 10% slowest RTs). Hence, to normalize the distributions, all data were log-transformed before analyses log_(1+ _*_x_*_)_.

Following this main analysis, we conducted linear regression analysis for examining associations between sex and all dependent variables. Because of the number of regressions performed, we adopted a conservative approach setting the threshold for statistical significance at 2-sided α = .01 for these analyses. Statistica software version 4.1 (StatSoft) was used to conduct analyses. Data analysis was performed from May to October 2020.

## Results

A total of 144 nurses (mean [SE] age, 41.3 [0.8] years; 92 women [63.9%]) were assigned to 1 of 2 groups according to the direction of shift rotation: 80 nurses (50 women) working an 8-hour fast FRS and 64 nurses (42 women) working an 8-hour fast BRS. As shown in Table 1, age, years of work experience, and sleep quality did not differ significantly between the shift rotation groups. The sex distribution did not differ between the groups (χ^2^_1_ = 0.15; *P* = .70). Hospitals working a FRS (Policlinico Umberto I, San Giovanni Addolorata, and Sant’Andrea) did not show significant differences on behavioral measures. Similarly, the remaining hospitals (San Carlo of Potenza and San Jacopo of Pistoia) adopting a BRS were not different.

**Table 1.** Demographic Variables and PSQI Scores

Variable	Mean (SE)		*t*_142_ ^a^	*P* value
	Forward rotating shift (n = 80)	Backward rotating shift (n = 64)		
Age, y	40.4 (1.0)	42.3 (1.3)	−0.89	.37
Time on the job, y	14.9 (0.9)	15.8 (1.4)	0.93	.35
PSQI subscale score				
Sleep duration	1.16 (0.07)	1.31 (0.09)	−1.22	.22
Sleep disturbance	1.35 (0.11)	1.17 (0.11)	0.91	.36
Sleep latency	1.11 (0.09)	0.94 (0.11)	1.34	.18
Daytime dysfunction	0.50 (0.10)	0.44 (0.09)	0.11	.91
Sleep efficiency	1.24 (0.06)	1.12 (0.06)	1.45	.15
Sleep quality	0.16 (0.07)	0.11 (0.07)	0.60	.55
Medication to sleep	1.00 (0.09)	1.12 (0.10)	−0.94	.35
Total score	6.54 (0.35)	6.22 (0.36)	0.80	.42

Abbreviation: PSQI, Pittsburgh Sleep Quality Index.

^a^ Data are the results of the *t* tests comparing the means log_(1 + _*_x_*_)_ values of the 2 groups.

The baseline characteristics related to the direction of shift rotation and sleep quality of the participants are outlined in Table 1. No statistically significant differences were found in the PSQI global score or sleep problem dimensions (ie, component scores) between the BRS and FRS groups. The mean total score of both groups exceeded the proposed cutoff of 5 for poor sleepers.^26^ Notably, 46 nurses (57.5%) in the FRS group and 37 nurses (57.8%) in the BRS group had poor sleep quality (χ^2^_1_ = 0.001; *P* = .97).

**Figure 1.**
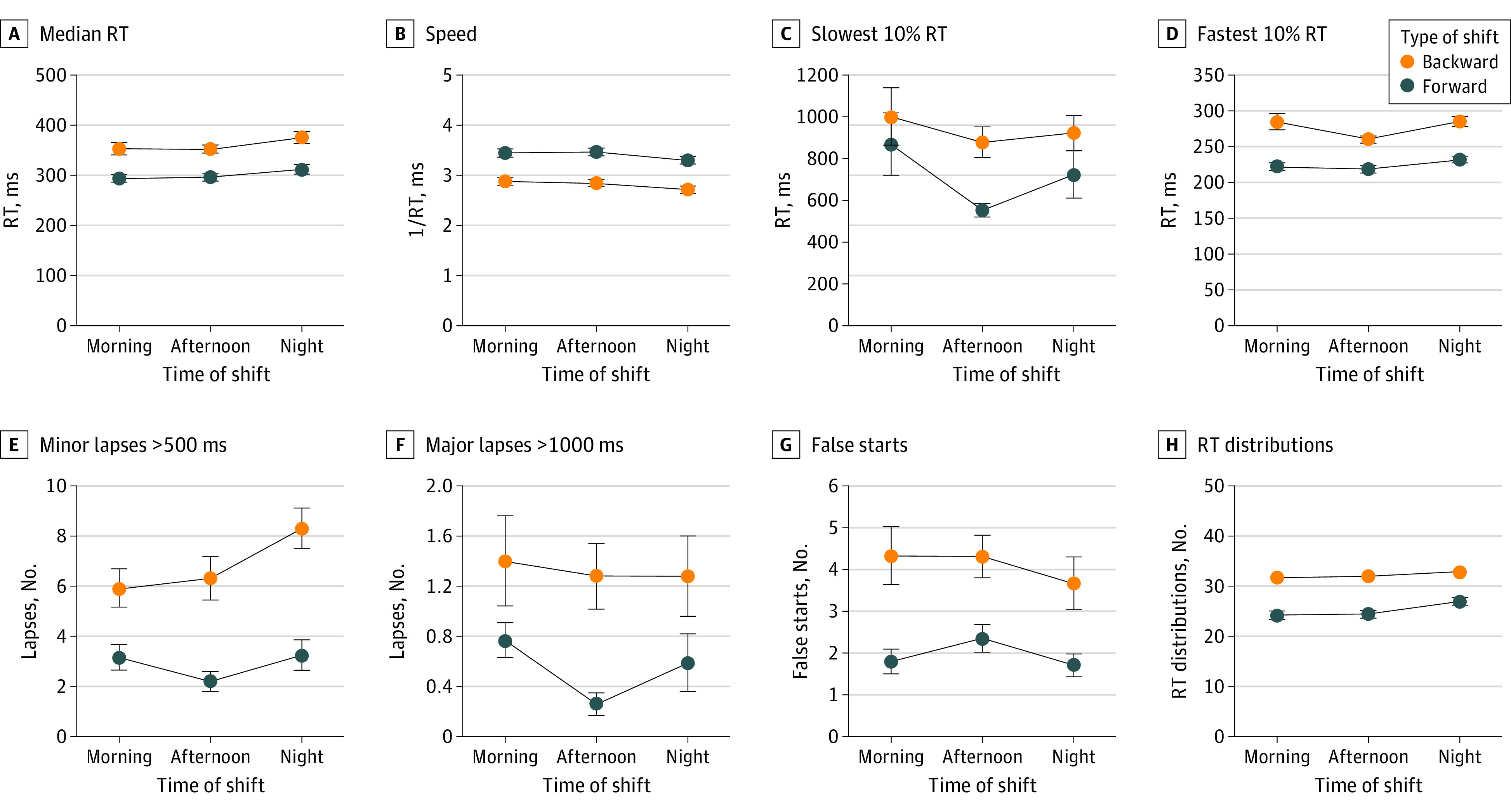
Psychomotor Vigilance Task Measures Data are means (dots) and SEs (error bars) for median reaction time (RT), speed (1 / RT), 10% slowest RT, 10% fastest RT, minor lapses (>500 milliseconds RT), major lapses (>1000 milliseconds RT), false starts, and RT distribution for nurses working in forward and backward shifts across different rapidly rotating shifts (morning, afternoon, and night).

**Figure 2.**
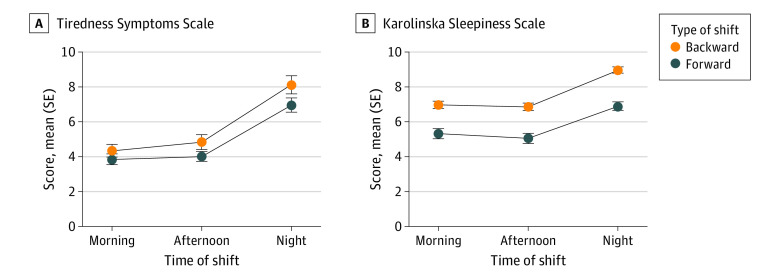
Tiredness Symptoms Scale Scores and Karolinska Sleepiness Scale Scores Data are mean (dots) and SE (error bars) scores, expressed as raw data, for nurses working in forward and backward shifts across different rapidly rotating shifts (morning, afternoon, and night).

Most importantly, there were significant differences between the BRS and FRS groups for all PVT variables (Table 2). BRS nurses had significantly worse attentional performance than those in the FRS group, as shown in Figure 1. BRS also demonstrated greater subjective sleepiness, as measured by the KSS (60 nurses working a BRS [93.8%] reported a KSS score of ≥7) compared with FRS (*F*_1,139_ = 41.23; *P* < .001), whereas the difference for tiredness, assessed by the TSS did not reach statistical significance (Figure 2 and Table 2).

**Table 2.** Results of the Statistical Analyses on Tiredness, Sleepiness, and Sustained Attention

Variable	Forward-rotating shift (n = 80), mean (SE)^a^			Backward-rotating shift (n = 64), mean (SE)^a^			Rotation		Shift		Rotation and shift interaction		Multivariate tests within-cell regression^b^	
	Morning	Afternoon	Night	Morning	Afternoon	Night	*F*_1,139_	*P* value	*F*_2,278_	*P* value	*F*_2,278_	*P* value	Wilks λ (Rao *R*_9,328_)	*P* value
TSS score	0.60 (0.03)	0.63 (0.03)	0.84 (0.03)	0.65 (0.04)	0.67 (0.04)	0.88 (0.04)	1.58	.21	67.91	<.001	0.02	.98	0.76 (4.38)	<.001
KSS score	0.76 (0.02)	0.73 (0.02)	0.88 (0.02)	0.89 (0.01)	0.88 (0.01)	0.99 (0.01)	41.23	<.001	43.29	<.001	0.59	.55	0.92 (1.30)	.23
PVT														
Median RT	2.46 (0.01)	2.47 (0.01)	2.49 (0.01)	2.54 (0.01)	2.54 (0.01)	2.57 (0.01)	42.12	<.001	7.78	<.001	0.06	.94	0.92 (1.17)	.31
Slowest 10%	2.80 (0.03)	2.71 (0.02)	2.75 (0.03)	2.89 (0.03)	2.86 (0.03)	2.89 (0.03)	13.77	<.001	3.26	.04	1.73	.18	0.94 (0.97)	.46
Fastest 10%	2.34 (0.01)	2.34 (0.01)	2.36 (0.01)	2.44 (0.01)	2.42 (0.01)	2.45 (0.01)	97.07	<.001	10.18	<.001	1.17	.31	0.96 (0.66)	.74
Minor lapses	0.45 (0.04)	0.35 (0.04)	0.43 (0.04)	0.69 (0.05)	0.73 (0.04)	0.85 (0.04)	46.29	<.001	4.37	.01	3.581	.02	0.93 (1.13)	.34
Major lapses	0.18 (0.02)	0.06 (0.02)	0.11 (0.02)	0.22 (0.04)	0.23 (0.04)	0.21 (0.04)	6.76	.01	2.79	.06	4.13	.02	0.92 (1.35)	.21
False starts	0.36 (0.03)	0.41 (0.03)	0.33 (0.03)	0.58 (0.05)	0.62 (0.04)	0.47 (0.05)	19.83	<.001	6.61	.002	0.96	.38	0.95 (0.82)	.59
RT distribution	1.37 (0.02)	1.39 (0.01)	1.43 (0.01)	1.51 (0.01)	0.51 (0.01)	1.53 (0.01)	60.13	<.001	8.88	<.001	2.73	.07	0.98 (0.24)	.99
Speed	0.64 (0.01)	0.65 (0.01)	0.63 (0.01)	0.59 (0.01)	0.58 (0.01)	0.57 (0.01)	56.90	<.001	8.80	<.001	0.28	.75	0.93 (1.13)	.34

Abbreviations: KSS, Karolinska Sleepiness Scale; PVT, Psychomotor Vigilance Task; RT, reaction time; TSS, Tiredness Symptoms Scale.

^a^ Data are log_(1 + _*_x_*_)_ values and results of the rotation (forward or backward) by shift (morning, afternoon, or night) analysis of covariance values of the dependent measures (TSS, KSS, and variables of the PVT), considering Pittsburgh Sleep Quality Index score, age, and years on the job as covariates.

^b^ Sex, Pittsburgh Sleep Quality Index scores, age, and years on the job were used as covariates.

Subjective tiredness and sleepiness were higher during the night shifts vs the morning shift (tiredness: *F*_1,143_ = 115.9; *P* < .001; sleepiness: *F*_1,143_ = 64.2; *P* < .001) and afternoon shift (tiredness: *F*_1,143_ = 92.0; *P* < .001; sleepiness: *F*_1,143_ = 76.7; *P* < .001) whereas the differences between morning and afternoon shifts were not significant (tiredness: *F*_1,143_ = 1.51; *P* = .23; sleepiness: *F*_1,143_ = 1.14; *P* = .29). We found that 51 nurses in the FRS group (63.7%) and 60 nurses in the BRS group (93.7%) reported very high levels of sleepiness at the end of their night shift (χ^2^_1_ = 18.12; *P* < .001). Therefore, the night shift was associated with almost all nurses (60 of 64) working backward in a state of elevated sleepiness. Night shifts were also associated with worse performance on the PVT: median RT (*F*_1,139_ = 42.12; *P* < .001), 10% fastest RT (*F*_1,139_ = 97.07; *P* < .001), minor lapses (*F*_1,139_ = 46.29; *P* < .001), and RT distribution (*F*_1,139_ = 60.13; *P* < .001) (Table 2). Specifically, the median RT was slower during night shifts than during morning and afternoon shifts (night vs morning: *F*_1,143_ = 10.47; *P* = .001; night vs afternoon: *F*_1,143_ = 10.89; *P* < .001), whereas the differences between morning and afternoon shifts were not significant (*F*_1,143_ = 0.35; *P* = .56). Furthermore, minor lapses and the RT distribution were characterized by similar differences, with more lapses and greater variability during night shifts than during morning (lapses: *F*_1,143_ = 4.31; *P* = .04; variability: *F*_1,143_ = 16.5; *P* < .001) and afternoon (lapses: *F*_1,143_ = 8.63; *P* = .004; variability: *F*_1,143_ = 15.01; *P* < .001) shifts, whereas the differences between morning and afternoon shifts were not significant (lapses: *F*_1,143_ = 0.80; *P* = .36; variability: *F*_1,143_ = 0.14; *P* = .72). Conversely, the 10% fastest RT was shorter during the afternoon shift than during the morning (*F*_1,143_ = 4.42; *P* = .03) and night (*F*_1,143_ = 4.52; *P* = .02) shifts. As detailed in Table 2, the rotation × shift interaction never reached significance, except for major and minor lapses.

The multivariate effect of covariates (PSQI scores, age, and years of work experience) was not significant, excepted for tiredness (Table 2). This finding indicated that PVT measures were significantly associated with TSS scores after each shift: morning (β = 0.40; 95% CI, 0.36-0.80; *P* < .001), afternoon (β = 0.41; 95% CI, 0.37-0.81; *P* < .001), and night (β = 0.39; 95% CI, 0.32-0.76; *P* < .001). Concerning the control of sex, the linear regressions examining the associations with all dependent variables mostly were not significant, except 4 variables of the PVT in the morning shift of the FRS condition (eTable in the Supplement).

## Discussion

This cohort study found that working fast BRSs was associated with increased sleepiness and decreased behavioral measures of sustained attention in nurses. Age, years of employment, and quality of sleep were not associated with modulating sleepiness and performance of this group. Notably, tiredness did not differ between the 2 groups.

Working schedules that conflict with the typical circadian rhythm are well-established to have negative effects. Physiologically, for a proper phase-shift in circadian alignment, shift rotation schedules should advance to allow easier adaption.^3^ The phenomenon is analogous to jet lag that occurs when traveling, when it is easier to adapt to traveling from east to west than from west to east, because eastward flights are associated with a phase advance of the internal clock.^34^

Contrary to our expectation, the 2 groups of shift workers did not differ in terms of any component of sleep quality, as measured using the PSQI. We found that 57.5% and 57.8% of the FRS and BRS groups, respectively, reported poor sleep quality (ie, score >5 on the PSQI), which is consistent with our previous studies (ie, 64%^21^ and 55%^20^) and studies in other countries (ie, 57%,^35^ 59%,^36^ 58%,^37^ 68.3%,^38^ 78%,^39^ and 61.9%^40^) and confirmed that night work is consistently associated with a high risk of sleep disruption and poor sleep quality.^41,42,43,44^

Conversely, the self-rated sleepiness of the BRS and FRS workers revealed a significant difference. Given that a KSS score of 7 or higher suggests a high degree of sleepiness, we found that 63.7% and 93.7% of nurses in the FRS and BRS groups, respectively, reported very high levels of sleepiness at the end of their night shift. Therefore, the night shift was associated with almost all nurses (60 of 64 nurses) working backward in a state of elevated sleepiness. Although these symptoms were elevated after the night, the BRS group also reported high sleepiness after morning and evening shifts, suggesting a stably elevated daytime sleepiness.

Another aspect of the difference between the 2 groups was that the BRS group reported higher sleepiness associated with shift rotation, which makes it more difficult to perform well.^45,46^ Indeed, we recorded the detrimental effects of anticlockwise rotation systems in health care on cognitive performance. The BRS groups had significantly slower speed, greater variability, more lapses and false starts, and longer lapse times than the FRS groups, with worse performance after night work than after day work. In 2016, an analysis estimated that medical errors are the third leading cause of death in the US.^47^ A medical error is a complex and multicausal phenomenon, in which sleepiness and impaired sustained attention are relevant components.^44^ Sleepiness has serious public health implications.^48^ Although, to our knowledge, there are no studies in a health care context, reduced sustained attention, as measured using the PVT, is associated with errors in workplaces.^49,50^ Therefore, strategies to reduce death from medical errors should also consider the adoption of a less disruptive shift-work schedule.^44,48^

### Limitations

This study has limitations. It is a field study, and we did not control for some potential confounders. For example, the BRS group performed more poorly on all outcome measures even after a rest day than the FRS group, and there were significant rotation × shift interactions limited to major and minor lapses. The current design does not allow us to disentangle 3 different interpretations: (1) the BRS group was unable to recover even after a rest day, (2) possible long-lasting consequences exist for working in BRSs, and (3) a fundamental difference exists between the groups that was not primarily due to the shift rotation direction. Furthermore, these interpretations are not necessarily mutually exclusive. Along this line, controlling of circadian and homeostatic factors was not possible in the current cohort study. The duration of the night shift was 11 hours, whereas the morning shift was 6.5 hours and the afternoon shift was 6 to 6.5 hours. However, these limitations are inherent in shift work. The typical comparison of morning, afternoon, and night shifts also intrinsically confounds 3 different factors: (1) homeostatic pressure (ie, the time spent after the last sleep period), (2) circadian phase, and (3) the sequence of days in the shift (ie, in a clockwise rotation system, afternoon and night shifts are the second and the third consecutive days on duty).

Ideally, the measures of tiredness (TSS), sleepiness (KSS), and cognitive slowing (PVT) should have been collected both before and after each shift, but we did not have the opportunity to collect these data. Similarly, we were not able to collect data on the participants’ habits (eg, use of caffeine, napping, or smoking) in some hospitals. This is another limitation of our study because these factors are likely to affect sleepiness and psychomotor vigilance. In fact, the increased coffee consumption during the night shift by BRS nurses has been reported as a potential countermeasure to sleepiness.^19^

## Conclusions

Considering the large number of different shift-work systems used worldwide, it is impossible to establish a single solution suitable for all workers. The current study compared a system consistent with circadian principles (ie, human circadian rhythms better tolerate phase delays than advances) vs a system with shorter recovery in the afternoon-morning shift sequence than in the morning-afternoon shift sequence (11 vs 24 hours), but longer recovery after the night shift (55 vs 48 hours). Our findings strongly discourage the adoption of BRS systems, favoring FRS work.

## Notes

Supplement. **eTable.** Results of the Linear Regressions for Examining Associations Between Sex and Dependent Variables
