# Supplementary material for: Comparison of Sleep and Attention Metrics Among Nurses Working Shifts on a Forward- vs Backward-Rotating Schedule
Source: JAMA Netw Open. 2021 Oct 18;4(10):e2129906. doi: 10.1001/jamanetworkopen.2021.29906 (PMC8524311; doi:10.1001/jamanetworkopen.2021.29906)
Supplement: Supplement. — eTable. Results of the Linear Regressions for Examining Associations Between Sex and Dependent Variables [file jamanetwopen-e2129906-s001.pdf]

Supplemental Online Content

Di Muzio M, Diella G, Di Simone E, et al. Comparison of sleep and attention metrics among nurses working shifts on a forward- vs backward-rotating schedule. *JAMA Netw Open*. 2021;4(10):e2129906. doi:10.1001/jamanetworkopen.2021.29906

eTable. Results of the Linear Regressions for Examining Associations Between Sex and Dependent Variables

This supplemental material has been provided by the authors to give readers additional information about their work.

© 2021 Di Muzio M et al. *JAMA Network Open.*

eTable. Results of the Linear Regressions for Examining Associations Between Sex and Dependent Variables

**FRS (N=80) (Females=1 Reference)**	**Morning**			**Afternoon**			**Night**		
	**B coefficient (95% CI)**	**T Statistic**	**P Value**	**B coefficient (95% CI)**	**T Statistic**	**P Value**	**B coefficient (95% CI)**	**T Statistic**	**P Value**
**TSS**	-0.154 (-2.153 to 0.393)	1.377	0.173	-0.137 (-1.877 to 0.450)	1.221	0.226	-0.126 (-2.645 to 0.738)	1.1222	0.265
**KSS**	-0.028 (-1.335 to 0.393)	0.246	0.807	-0.045 (-1.453 to 0.973)	0.394	0.695	-0.202 (-1.900 to 0.086)	1.818	0.073
**PVT**									
**Median RT**	**-0.347 (-66.65 to -16.20)**	**3.270**	**0.002**	-0.214 (-48.50 to 0.67)	1.937	0.06	-0.120 (-56.72 to 17.01)	1.072	0.29
**Slowest 10%**	-0.100 (-888.90 to 340.34)	0.888	0.38	-0.215 (-256.57 to .05)	1.944	0.06	-0.153 (-781.73 to 145.74)	1.365	0.18
**Fastest 10%**	**-0.321 (-31.70 to -6.37)**	**2.992**	**0.004**	-0.164 (-21.28 to 3.21)	1.470	0.15	-0.152 (-25.05 to 4.74)	1.357	0.18
**Minor lapses**	-0.166 (-3.65 to 0.53)	1.488	0.14	-0.117 (-2.49 to 0.79)	1.04	0.30	0.002 (-2.49 to 2.54)	0.021	0.98
**Major lapses**	-0.090 (-0.79 to 0.34)	0.795	0.43	-0.185 (-0.69 to 0.06)	1.667	0.10	-0.094 (-1.37 to 0.56)	0.836	0.41
**False starts**	-0.127 (-1.48 to 0.41)	1.127	0.26	-0.117 (-2.09 to 0.65)	1.043	0.30	0.064 (-0.80 to 1.44)	0.570	0.57
**RT distribution (RTD)**	**-0.328 (-8.461 to -1.80)**	**3.071**	**0.003**	-0.191 (-5.68 to 0.44)	1.707	0.10	-0.231 (-5.86 to -0.15)	0.209	0.04
**Speed**	**0.371 (0.19 to 0.68)**	**3.52**	**0.001**	0.246 (0.03 to 0.49)	2.246	0.03	0.174 (-0-05 to 0.42)	1.565	0.12

© 2021 Di Muzio M et al. *JAMA Network Open.*

**BRS (N=64) (Females=1 Reference)**	**Morning**			**Afternoon**			**Night**		
	**B coefficient (95% CI)**	**T Statistic**	**P Value**	**B coefficient (95% CI)**	**T Statistic**	**P Value**	**B coefficient (95% CI)**	**T Statistic**	**P Value**
**TSS**	-0.178 (-2.59 to 0.43)	1.426	0.163	-0.136 (-2.79 to 0.83)	1.085	0.28	-0.094 (-3.00 to 1.37)	0.744	0.46
**KSS**	0.273 (0.10 to 1.80)	2.236	0.03	0.079 (-0.63 to 1.20)	0.621	0.54	-0.171 (-1.24 to 0.23)	1.371	0.17
**PVT**									
**Median RT**	0.084 (-35.09 to 70.23)	0.667	0.51	0.059 (-22.80 to 36.58)	0.464	0.64	0.194 (-10.61 to 85.67)	1.558	0.12
**Slowest 10%**	0.161 (-205.88 to 947.33)	1.285	0.20	0.072 (-223.88 to 400.68)	0.566	0.57	0.036 (-304.19 to 404.72)	0.283	0.78
**Fastest 10%**	-0.129 (-71.56 to 23.16)	1.022	0.31	0.104 (-8.49 to 0.40)	0.824	0.41	-0.122 (-45.83 to 15.94)	0.967	0.34
**Minor lapses**	0.104 (-1.90 to 4.58)	0.828	0.41	0.124 (-1.43 to 4.22)	0.985	0.33	0.066 (-2.57 to 4.39)	0.522	0.60
**Major lapses**	0.140 (-0.66 to 2.33)	1.116	0.27	0.124 (-0.56 to 1.64)	0.980	0.33	0.889 (-0.87 to 1.82	0.701	0.49
**False starts**	0.267 (0.23 to 5.28)	2.180	0.03	0.042 (-1.80 to 2.51)	0.329	0.74	0.81 (-1.85 to 3.59)	0.639	0.52
**RT distribution (RTD)**	-0.146 (-3.10 to 0.82)	1.163	0.25	0.068 (-1.48 to 2.58)	0.539	0.59	-0.126 (-2.85 to 0.95)	1.000	0.32
**Speed**	-0.182 (-0.41 to 0.06)	1.460	0.15	-0.072 (-0.30 to 0.17)	0.571	0.57	-0.080 (-3.09 to 0.16)	0.631	0.53

Results of the linear regression for examining associations between sex and all dependent measures (Tiredness Symptoms Scale [TSS], Karolinska Sleepiness Scale [KSS], variables of the psychomotor vigilance task [PVT]). Significat regressions (α = 0.01) have been reported in bold.

© 2021 Di Muzio M et al. *JAMA Network Open.*
